# Prevalence of *Escherichia coli O157:H7* Bacterial Infections Associated With the Use of Animal Wastes in Louisiana for the Period 1996–2004

**DOI:** 10.3390/ijerph2006030012

**Published:** 2006-03-31

**Authors:** Dagne D. Hill, William E. Owens, Paul B. Tchounwou

**Affiliations:** 1 Department of Biological Sciences, Grambling State University, P.O. Box 887, Grambling, Louisiana, USA; 2 Louisiana State University Agricultural Experiment Station, Hill Farm Research Station, 11959 Highway 9, Homer, Louisiana, USA; 3 Molecular Toxicology Research Laboratory, NIH-Center for Environmental Health, College of Science, Engineering and Technology, Jackson State University, 1400 Lynch Street, P.O. Box 18540, Jackson, Mississippi, USA

**Keywords:** Non-point source pollution, *E. coli* O157:H7, Runoff water, Land application

## Abstract

Animal waste from dairy and poultry operations is an economical and commonly used fertilizer in the state of Louisiana. The application of animal waste to pasture lands not only is a source of fertilizer, but also allows for a convenient method of waste disposal. The disposal of animal wastes on land is a potential non-point source of water degradation. Human health is a major concern when considering the disposal of large quantities of animal waste. Health concerns could exist from exposure to pathogens and excess nitrogen associated with this form of pollution. The objective of this study was to collect and analyze health data related to *Escherichia coli O157:H7* bacterial infections associated with the use of animal waste in Louisiana for the years 1996–2004. An analysis of adverse health effects associated with the use of animal waste in Louisiana was conducted based on the incidence/prevalence rate for the studied years. The number of reported cases increased during the summer months. Analysis of health data of the studied years showed that the number of reported disease cases of *E. coli* O157:H7 were highest among Caucasian infants in the 0–4 year old age category and in Caucasian children in the 5–9 year old age category. Although the number of cases declined with age, a slight increase in rates was seen among the elderly population. While the rate of reported cases per 100,000 people remained the same for the years of 1999 and 2000, the rate decreased by 60% from the year 2000 to 2001. A slight decline of the number of cases that was also reported for the years 2002 and 2003. The high rate of identification in the younger population may result from the prompt seeking of medical care when symptoms become evident among infants and young children as well as the frequent ordering of stool examination when symptoms become evident in this population group. It was also noted that areas that had a higher number of reported cases also had a greater number of physicians per 100,000 people within the parish. The association with increasing age could be attributed to declining health and weaker immune systems often found among the older population. It was concluded that although some of the studied parishes surveyed had large amounts of animal waste generated each year, statistics did not show a correlation with *Escherichia coli O157:H7* bacterial infections.

## Introduction

The application of animal waste to pasture lands not only is a source of fertilizer, but also allows for a convenient method of waste disposal. One type of animal waste product that is commonly used is dairy lagoon sediment or effluent. Many dairies in Louisiana have a one or two stage lagoon that collects liquid and semi-liquid manure from loafing barns and milking parlor areas. The solid waste settles in the lagoon where it is reduced by the process of anaerobic digestion. The liquid effluent is often pumped onto fields or is recycled for other uses. Periodically, the lagoon must be emptied of the sediment build up. The sediment is typically agitated in order to suspend it into a semi-liquid state and is pumped onto the fields. The disposal of animal wastes on land is a potential non-point source of water degradation. Runoff and percolation could possibly transport organic matter, bacteria and nutrients to surface and ground water. Animal wastes applied to the land come from wastes that have been removed from feeding facilities, runoff from feeding areas, and waste from animals on pasture and rangeland. The proper application of animal wastes can not only provide nutrients for crop production, but can also reduce surface runoff bacterial exposure.

The health of humans is of concern when considering large quantities of animal waste. Some of the main concerns include the exposure to pathogens and excess nitrogen associated with this form of pollution. Animal waste can contain pathogens, such as faecal coliform bacteria and viruses that may contaminate drinking water and may cause gastrointestinal illnesses. High levels of nitrogen leaching into drinking water supplies can increase the risk of methemoglobinemia [1]. In 1996, the Centers for Disease Control linked the high nitrate levels in Indiana well water near feedlots to spontaneous abortions in humans [2].

Nitrites are relatively short-lived because they’re quickly converted to nitrates by bacteria. Nitrites produce a serious illness (brown blood disease) in fish, even though they don’t exist for very long in the environment. Nitrites also react directly with hemoglobin in human blood to produce methemoglobin, which destroys the ability of blood cells to transport oxygen. This condition is especially serious in babies under three months of age as it causes a condition known as methemoglobinemia or “blue baby” disease. Water with nitrite levels exceeding 1.0 mg/L should not be given to babies. Nitrite concentrations in drinking water seldom exceed 0.1 mg/L [3].

Nitrate is a major ingredient of farm fertilizer and is necessary for crop production. When it rains, varying nitrate amounts wash from farmland into nearby waterways. Nitrates also get into waterways from lawn fertilizer run-off, leaking septic tanks and cesspools, manure from farm livestock, animal wastes, and discharges from car exhaust. Nitrates can be reduced to toxic nitrites in the human intestine. The U.S. Public Health Service has established 10 mg/L of nitrate-nitrogen as the maximum contamination level allowed in public drinking water [3]. Nitrate-nitrogen levels below 90 mg/L and nitrite levels below 0.5 mg/L seem to have no effect on warm-water fish, but many cold water fish are more sensitive. The recommended nitrite minimum for salmon is 0.06 mg/L [4].

Ammonia is a toxic form of nitrogen. Open air lagoons emit ammonia into the air [5]. One survey of residents living in the vicinity of a 2,500-sow facility found much higher reports of respiratory problems than were recorded from the neighbourhoods of farms where no livestock was raised [6].

Many regulations for water are found in the Clean Water Act. The H.R. 961, a bill to reauthorize the Clean Water Act, was approved by the House of Representatives. This bill provides program authority and funding for Fiscal Years 1996 through 2000. The H.R. 961 bill would reverse a 1994 Federal circuit court ruling that land application of livestock manure from a concentrated animal feeding operation is a point source which is subject to permit and enforcement provisions of the CWA (*Concerned Area Residents for the Environment v. Southview Farm,* No. 93-9229 {2 Cir. Sept. 2, 1994}). The Supreme Court recently declined review of the *Southview Farm* case [7].

Drinking water quality has been improving over time. According to the Centers for Disease Control and Prevention (CDC), the proportion of reported disease outbreaks that can be attributed to problems at public water treatment systems has steadily declined, from 73% in 1989 – 1990 to 30% in 1995 – 1996. It is possible that this decrease reflects the improvements in water treatment and in operation of plants [8].

Between the years of 1997 and 1998, 13 states reported a total of 17 significant illness outbreaks associated with drinking water. The CDC keeps records on occurrences and causes of outbreaks of illness related to drinking water and recreational water. Man y outbreaks are often missed by public health officials because some illnesses that are associated with the outbreaks are not perceived as being water related [9]. The Surveillance Summaries of the Morbidity and Mortality Weekly Reports noted that between the years of 1997 and 1998, there were four outbreaks caused by bacteria; three were attributed to *E. coli* O157:H7 and one to *Shigella sonnei.* One of these outbreaks occurred in the state of Illinois and involved three persons who drank from an untreated well located near a cattle pasture. Another outbreak involving 26 people was noted to have occurred in the state of Georgia at a water park. It is believed that a fecal accident in the children’s wading pool was the source. Nine persons became ill from *Shigella sonnei* in Massachusetts. This outbreak was associated with a wading pool that included a sprinkler fountain and was used by many diaper-aged children [9].

In 1999, according to the New York Times, *E. coli* contaminated water at the Washington County fairgrounds in New York State caused the death of two people and illness in over 1000 others. The source of contamination was probably cattle fecal material from a nearby barn, which was swept into the soil by storm runoff, and then leached into the aquifer [10].

Drinking water health effects are not limited to gastrointestinal illness associated with microbes. Drinking water can transmit bacteria, micro-organisms, and chemicals that are capable of causing disease. The symptoms can be acute, such as diarrhoea and dehydration, or they can be long term effects that include infertility and reproductive health effects, or chronic illnesses such as cancer [11].

## Materials and Methods

### Method

An analysis of the number of cases and the incidence rate per 100,000 people of *E. coli O157:H7* was conducted for each of the 64 parishes located in Louisiana. The analysis included the years 1996–2004. This study utilized materials received from Louisiana Health and Hospital Systems, Infectious Disease Epidemiology Section, New Orleans, LA. Patterns in regards to race, age and gender were recorded. The parishes with high incidence rates of these illnesses were identified. Data from the 2000 census retrieved from the United States Census Bureau were also utilized in order to identify populations at risk so that comparisons of disease incidence rates by parish, area, or other characteristics could be made. An analysis of the total amounts of animal waste, cattle waste, poultry waste, amounts of nitrogen in waste and the amount of phosphorus in waste was also analyzed from data retrieved from Environmental Defense and GetActive Software.

## Statistical Analysis

Demographic data and data on the numbers of cases of *E. coli* infections in Louisiana were collected. Cases of diseases were divided by the population at risk to determine the incidence/prevalence rates. These disease rates were used as the basis or end-point for comparing the health risks by parishes, gender, race, etc. Additional information to what was already mentioned was collected on the amounts of animal wastes generated by parish.

A linear regression analysis was performed to determine if there is a correlation between the tons of cattle waste and the incidence of diseases by parish as well as the correlation between the tons of poultry waste and the incidence of diseases by parishes.

## Results

An analysis of *E. coli* O157:H7 cases for the state of Louisiana showed that for the years 1996–2001, the highest reported cases were in 1997 followed by the year 2000.

**Figure 1:**
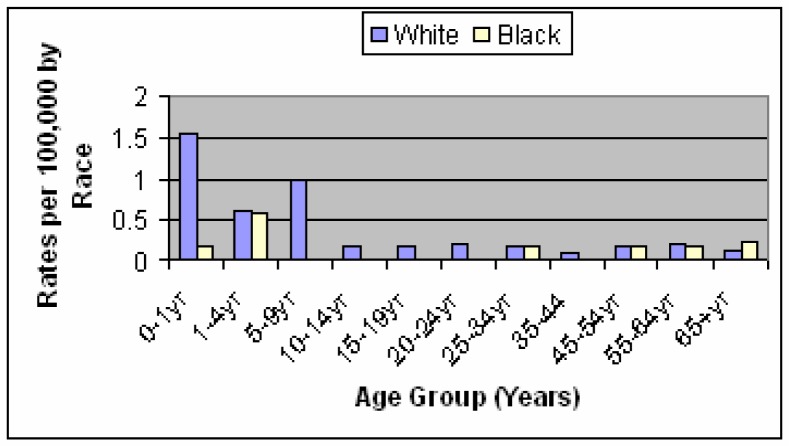
*E.coli* O157:H7 average incidence rates by race and age for the period 1996 – 2001 in Louisiana (LDHH 2001).

Figure 1 shows that of the period 1996–2001, the majority of the cases were reported for young Caucasian infants and children between the ages of 0 and 9 years of age. The highest numbers of cases reported during the years of 1996–2001 were found in St. Tammany Parish with a total of 11 cases [13].

**Figure 2:**
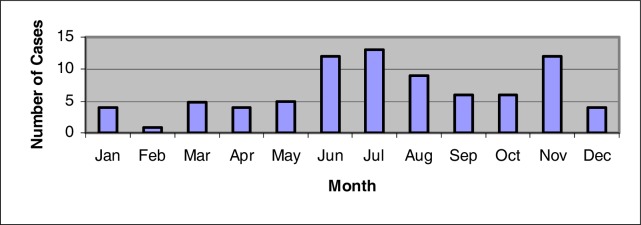
Average annual cases of *E.coli* O157:H7 by seasonal distribution for the period 1996 – 2001 in Louisiana (LDHH 2001).

Figure 2 shows that the *E. coli* O157:H7 number of cases reported increased during the months of June, July, August and November. These months reported 12, 13, and 9 cases, respectively. The fewest number of reported cases during the years of 1996–2001, according to Figure 2, was during the month of February. During 1999, sixty-four percent of the cases reported occurred between the months of June and September [12]. For the years of 1996–2001, 1 case was reported during the month of February [13].

Table 1 compares incidence rates that were greater than 2 per 100,000 people for the years 1999–2004. It was noted that there were more parishes that had an incidence rate greater than 2 per 100,000 people in 1999, according to Table 1, than any of the other compared years. In 1999, as indicated in Table 1, the highest reported rate was for Richland Parish. Richland Parish had a rate of 14.3 per 100,000 people. Other parishes that had a rate that was greater than 2 per 100,000 people in that same year included Washington, Avoylles, Assumption and St. James. The incidence rates that were reported for these parishes were 2.28, 2.41, 4.28, and 9.43, respectively. It was further noted that Washington Parish was the only studied parish that had this high incidence rate for more than one year [14].

**Figure 3:**
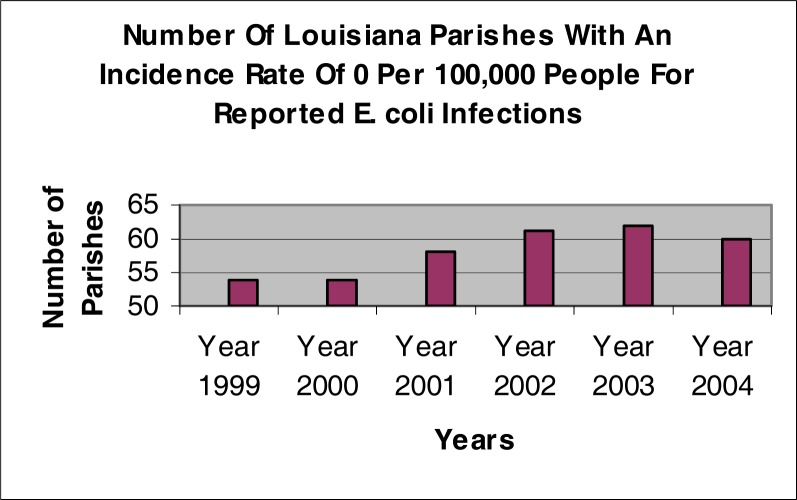
Number of Louisiana parishes with an incidence rate for *E.coli* O157:H7 of 0 per 100,000 people for years 1999 – 2004 in Louisiana (LDHH 2005)

Figure 3 indicates that the number of parishes that reported an incidence rate of 0 per 100,000 people increased from 54 parishes in 1999 to 62 parishes in 2003. A slight decrease in the reported number of parishes is shown in 2004 with only 60 parishes being reported with an incidence rate of 0 per 100,000 people.

**Figure 4:**
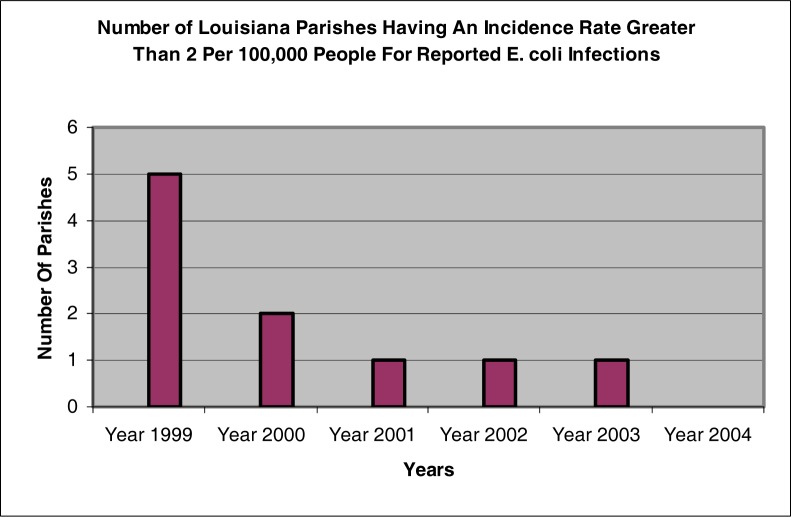
Number of Louisiana parishes with an incidence rate for *E. coli* O157:H7 greater than 2 per 100,000 people for years 1999 – 2004 in Louisiana (LDHH 2005)

Figure 4 indicates that during 1999, 5 parishes had an incidence rate greater than 2 per 100,000 people. A comparison of Tables 2, 3, and 4 show a decrease in the number of parishes with an incidence rate greater than 2 per 100,000 people. These tables indicate a reporting of 3 parishes in 1999 and a reporting of only 1 parish in 2001. Tables 5 and 6 both indicate that only 1 parish had an incidence rate greater than 2 per 100,000 people for the years 2002 and 2003.

Using the described statistical methods, no apparent significant correlation (p>0.05) between the amount of animal waste and the studied disease incidence rates among the parishes in the state of Louisiana was observed.

As indicated in Table 7 and figure 4, none of the parishes reported an incidence rate greater than 2 per 100,000 people in 2004.

## Discussion

The state of Louisiana is reported as having a total of 7,876,528 acres of farmland. Of this amount, Washington Parish has a total of 100,006 acres [15].

*E. coli* O157:H7 is found in both dairy and beef herds in the majority of cattle farms across the United States [12]. Infection from this organism is considered as a Class B disease and must be reported to the state within one business day. It became reportable in Louisiana in 1996, with the number of cases ranging between five to twenty cases per year. The detection is higher among infants than among children and adults because infants with diarrhoea are more likely to be brought to a medical facility to have stool examination. A higher number of cases are reported for Caucasian than for African Americans. A possible explanation for the low number of African Americans reporting the disease is possibly the result of a lack of access to medical care for this community. Because of this, more screening is done for Caucasian than for African Americans. According to the Louisiana Department of Health and Hospitals, the average number of primary care physicians per 10,000 in population is 10. St. Landry Parish, which had an incidence rate greater than 2 per 100,000 people in the year 2000, has an average of 9 primary care physicians. The parish of Washington has an average of 7 primary care physicians. Both St. Landry and Washington Parish have a total population of less than 100,000 residents [19]. Another possible explanation to the differences in the amount of cases reported between the Caucasian and African American community could be that the parishes reporting the highest rates of this illness also had a much lower minority population as compared to the majority population [12]. The highest rate among many of the studied diseases was not only among Caucasian, but in infants in the 0–4 year old age category and in children in the 5–9 year old age category. The largest segment of the population in Red River and Washington Parishes was found to be in the under 18 year old age group. This probably accounts for such large numbers of young residents being reported [13]. As mentioned earlier, a factor as to the possibility of why this segment of the population is represented in such high numbers is the possibility that Washington Parish also had more access to health care and more screening by health officials in which the disease would have been required to be reported. This would be expected in a parish like St. Tammany in which 80.66% of the residents were high school graduates or higher. Greater than 50% of residents for many Louisiana parishes have a high school diploma or above. There is no correlation seen among the parishes when comparing the educational status of the residents to the percent of families living below the poverty level in 1999 [15]. In regards to the high number of infants and children being infected with the studied diseases, the risk from direct contact with faecal material at farms and petting zoos is also recognized as an important factor [17].

Many of the surface water areas Louisiana parishes include estuaries. Estuaries can become contaminated with fecal coliform bacterial pollution as a result of rainfall runoff from urbanized areas [18].

In 1997, as seen in Table 8, Washington Parish ranked second in the state among the sixty-four parishes with 490,000 tons of cattle waste and ranked thirteenth with regards to poultry waste generated, with only 64 tons. In 1997, a total of 5,300,000 pounds per year of nitrogen in animal waste and a lower amount of 1,200,000 pounds per year of phosphorous in animal waste were reported for Washington Parish [16].

Compared to the other parishes in the state, Washington Parish ranked fourth in the amount of phosphorus reported in animal waste and third in the amount of nitrogen reported in animal waste. Of the reported amount of nitrogen, 1,600,000 pounds per year was lost to the atmosphere. Although Assumption Parish had a reported incidence rate greater than 2 per 100,000 people, as shown in Table 1, it also was found, as shown in Table 8, to have the lowest amount in tons of animal waste generated of any of the other parishes within the state of Louisiana [16]. There was no correlation found to exist between the amount of animal waste stored within the parishes and the incidence rates of the studied disease. It must also be noted that the amounts of animal waste totals reported from the Environmental Defense and GetActive Software seem relatively high based upon the number of animals used in the calculation.

## Conclusions

Survey of literature information regarding *E. coli* O157:H7 infections within the state of Louisiana would seem to indicate that this disease has an animal association. It is possible that some of the cases were related to animal waste that is used for the purpose of fertilization, but there is no clear indication that any cases have this origin or association. Although some of the studied parishes surveyed had large amount of animal waste generated each year, statistics do not show a correlation between this and the studied disease.

## Figures and Tables

**Table 1:** Infection rates greater than 2/100,000 people for *E. COLI O157:H7* in Louisiana parishes (LDHH 1999–2005)

*Year*	*Parish Name*				
1999	Washington 2.28*	Avoylles 2.41*	Assumption 4.28*	St. James 9.43*	Richland 14.3*
2000	St. Landry 2.28*	St. Charles 4.16*			
2001	Washington 4.55*				
2002	Jackson 6.49*				
2003	Union 4.39*				
2004	None				

* Rates

**Table 2:** Distribution of *E. coli* O157:H7 in various parishes for the year 1999

*Group*	Rate*	*No. of Parishes*
A	0.00	54
B	0.01–1.00	3
C	1.01–2.00	2
D	2.01–3.00	2
E	>2.00	3

* Number of cases per 100,000 people

**Table 3:** Distribution of *E. coli* O157:H7 in various parishes for the year 2000

*Group*	Rate*	*No. of Parishes*
A	0.00	54
B	0.01–1.00	5
C	1.01–2.00	3
D	>2	2

* Number of cases per 100,000 people

**Table 4:** Distribution of *E. coli* O157:H7 in various parishes for the year 2001

*Group*	Rate*	*No. of Parishes*
A	0.00	58
B	0.01–1.00	5
C	1.01–2.00	0
D	>2	1

* Number of cases per 100,000 people

**Table 5:** Distribution of *E.coli* O157:H7 in various parishes for the year 2002

*Group*	Rate*	*No. of Parishes*
A	0.00	61
B	0.01–1.00	1
C	1.01–2.00	1
D	>2	1

* Number of cases per 100,000 people

**Table 6:** Distribution of *E.coli* O157:H7 in various parishes for the year 2003

*Group*	Rate*	*No. of Parishes*
A	0.00	62
B	0.01–1.00	0
C	1.01–2.00	1
D	>2	1

* Number of cases per 100,000 people

**Table 7:** Distribution of *E.coli* O157:H7 in various parishes for the year 2004

*Group*	Rate*	*No. of Parishes*
A	0.00	60
B	0.01–1.00	2
C	1.01–2.00	2
D	>2	0

* Number of cases per 100,000 people

**Table 8:** Amount of cattle and poultry waste for Louisiana parishes (Environmental Defense and Get Active Software 2003)

*Parish*	*Cattle Waste Amount (tons)*	*Rank*	*Poultry Waste Amount (tons)*	*Rank*
Tangipahoa	680000	1	96	12
*Washington*	490000	2	64	13
*Desoto*	360000	3	2	38
Bossier	250000	4	20	25
Calcasieu	250000	5	27	22
Vermilion	240000	6	37	21
Natchitoches	240000	7	24,000	6
Beauregard	200000	8	45	16
*Red River*	200000	9	0	54
St. Landry	190000	10	52	14
Cameron	190000	11	0	61
Rapides	170000	12	42	17
St. Helena	170000	13	0	51
East Feliciana	160000	14	0	58
LaFourche	160000	15	41	18
Avoyelles	140000	16	38	20
Pointe Coupee	140000	17	0	55
Caddo	130000	18	1	44
East Baton Rouge	130000	19	120	11
Jefferson Davis	130000	20	11	31
Franklin	130000	21	10	33
Union	130000	22	250,000	1
West Carroll	110000	23	13	30
Sabine	110000	24	120,000	2
Iberville	110000	25	0	46
Vernon	110000	26	13,000	7
Evangeline	100000	27	14	28
Claiborne	96000	28	47,000	5
Richland	89000	29	2	41
Catahoula	85000	30	0	62
Lafayette	83000	31	45	16
Lincoln	78000	32	77,000	3
Ouachita	78000	33	12,000	8
Grant	74000	34	18	26
Webster	74000	35	0	49
Allen	71000	36	1	43
West Feliciana	70000	37	0	48
Acadia	65000	38	21	23
St. Tammany	65000	39	39	19
Ascension	62000	40	13	29
Livingston	59000	41	7,800	9
Winn	59000	42	0	47
Bienville	58000	43	5,300	10
Morehouse	56000	44	2	42
LaSalle	56000	45	0	56
Plaquemines	47000	46	7	34
Caldwell	44000	47	21	24
Terrebonne	33000	48	2	39
Iberia	32000	49	15	27
Concordia	32000	50	0	60
East Carroll	29000	51	0	59
St. Charles	28000	52	0	52
Jackson	26000	53	52,000	4
St. Martin	25000	54	11	32
Madison	22000	55	2	40
Jefferson Davis	14000	56	11	31
St. Mary	12000	57	4	36
West Baton Rouge	11000	58	4	35
Tensas	7200	59	0	37
St. Bernard	6600	60	0	53
St. John The Baptist	4100	61	0	50
Assumption	3200	62	0	45
